# MED13/378: Using the Internet to Learn about Health Care Systems: The Health care game

**DOI:** 10.2196/jmir.1.suppl1.e56

**Published:** 1999-09-19

**Authors:** J Westbrook, J Braithwaite, M Chaloupka, A Lovell-Simons

**Affiliations:** ^1^University of Sydney, LidcombeAustralia

**Keywords:** Education, Internet, Health Care

## Abstract

Health science students often struggle with the complexity and diversity of the Australian health care system. They tend to view the system from their own frame of reference and not through the frame of others. To address these learning challenges we designed a web-based heuristic game which promotes information seeking skills and provides a multi-frame approach to teaching and learning. The Health Care Game is accessed by students via the Internet and comprises four hypothetical families from different cultural, socio-economic and demographic backgrounds. Family members encounter a series of health events during the course of the game. Each event generates problems for the family member. Students are required to obtain information which will allow the family to navigate the health system, identify the services required, access and obtain the cost of these services. Options developed by students for the family need to consider the family's background including available financial resources. Internet resources support students' information seeking activities and several sites are linked to the game. Key features of the game include collaboration in small groups in competition with other groups, and online discussion and feedback facilities. The course co-ordinator has the ability to monitor the online discussion sessions of teams without interfering with group dynamics. If teams are having difficulty the course co-ordinator can provide guidance by e-mail. Teams seek advice from the course co-ordinator in the same way. The game is based upon a dynamic database of health events which may be edited, ensuring events are relevant and allowing the game to be tailored to different health science courses. The game is currently used by first year students undertaking a Bachelor of Applied Science (Health Information Management) at the University of Sydney, Australia. A three-stage evaluation process of the game will be completed in July 1999. Implementation of the game within a university environment has highlighted the need for appropriate IT support structures for academics who wish to use internet resources as an integral part of the curricula. This includes developing strategies by which learning technologists, programmers and academic staff can communicate with each other, as they too, proceed from differing frames of reference from each other. This project was supported by a National Teaching Development Grant (CAUT).

